# Cerebrospinal fluid α-synuclein predicts neurodegeneration and clinical progression in non-demented elders

**DOI:** 10.1186/s40035-020-00222-1

**Published:** 2020-11-23

**Authors:** Jie-Qiong Li, Yan-Lin Bi, Xue-Ning Shen, Hui-Fu Wang, Wei Xu, Chen-Chen Tan, Qiang Dong, Yan-Jiang Wang, Lan Tan, Jin-Tai Yu, Alzheimer’s Disease Neuroimaging Initiative

**Affiliations:** 1 Department of Neurology and Institute of Neurology, Huashan Hospital, Shanghai Medical College, Fudan University, Shanghai, 20040 China; 2 Department of Neurology, Qingdao Municipal Hospital, Qingdao University, Qingdao, 266071 China; 3 Department of Neurology, The Affiliated Hospital of Qingdao University, Qingdao, 266071 China; 4 Department of Anesthesiology, Qingdao Municipal Hospital, Qingdao University, Qingdao, 266071 China; 5 Department of Neurology and Center for Clinical Neuroscience, Daping Hospital, Third Military Medical University, Chongqing, 400042 China

**Keywords:** Alzheimer’s disease, α-Synuclein, Biomarker, Cerebrospinal fluid, Neurodegeneration

## Abstract

**Background:**

Accumulating reports have suggested that α-synuclein is involved in the pathogenesis of Alzheimer’s disease (AD). As the cerebrospinal fluid (CSF) α-synuclein has been suggested as a potential biomarker of AD, this study was set out to test whether CSF α-synuclein is associated with other AD biomarkers and could predict neurodegeneration and clinical progression in non-demented elders.

**Methods:**

The associations between CSF α-synuclein and other AD biomarkers were investigated at baseline in non-demented Chinese elders. The predictive values of CSF α-synuclein for longitudinal neuroimaging change and the conversion risk of non-demented elders were assessed using linear mixed effects models and multivariate Cox proportional hazard models, respectively, in the Alzheimer’s disease Neuroimaging Initiative (ADNI) database.

**Results:**

The CSF α-synuclein levels correlated with AD-specific biomarkers, CSF total tau and phosphorylated tau levels, in 651 Chinese Han participants (training set). These positive correlations were replicated in the ADNI database (validation set). Using a longitudinal cohort from ADNI, the CSF α-synuclein concentrations were found to increase with disease severity. The CSF α-synuclein had high diagnostic accuracy for AD based on the “ATN” (amyloid, tau, neurodegeneration) system (A + T+ versus A − T − control) (area under the receiver operating characteristic curve, 0.84). Moreover, CSF α-synuclein predicted longitudinal hippocampus atrophy and conversion from MCI to AD dementia.

**Conclusions:**

CSF α-synuclein is associated with CSF tau levels and could predict neurodegeneration and clinical progression in non-demented elders. This finding indicates that CSF α-synuclein is a potentially useful early biomarker for AD.

**Supplementary Information:**

The online version contains supplementary material available at 10.1186/s40035-020-00222-1.

## Background

Alzheimer’s disease (AD) is the leading cause of dementia in the elderly and is clinically characterized by a gradual decline in memory and other cognitive functions. However, less than half of the patients with dementia have received a formal diagnosis in Europe and the USA [1]. The pathological change of AD can precede the onset of clinical symptoms by 20 years. Biomarker research has made it possible to identify people at the high risk of developing dementia in the general population, even at the preclinical stage [2, 3]. According to the newly published “ATN” scheme, various biomarkers can be divided into three binary components: (i) biomarkers of β-amyloid (Aβ) plaques or associated pathophysiologic processes labeled as “A”; (ii) biomarkers of aggregated pathologic tau or associated pathophysiologic processes labeled as “T”; and (iii) biomarkers of neurodegeneration or neuronal injury labeled as “N” [4]. Besides the biomarkers mentioned above, additional novel biomarkers that reflect other disease mechanisms may provide insights into the different mechanisms of AD pathogenesis and assist in identifying novel therapeutic targets in the future. This was echoed by the 2018 NIA-AA research framework that “ATN” can be expanded to incorporate other proteinopathies that are also involved in AD pathogenesis or frequently co-occur with AD pathologic changes [5–7]. This provided a multidimensional approach to diagnosing dementia and for better clinical stratification of patients for therapeutic trials [8, 9].

α-Synuclein is best known for its roles in Parkinson’s disease (PD) and dementia with Lewy bodies (DLB), and has also been reported to be implicated in AD pathogenesis [10]. Patients with AD and concomitant α-synuclein pathology typically have a more rapid rate of cognitive decline than those with AD alone [11, 12]. α-Synuclein is generally considered as a pre-synaptic protein, which can also be found in human cerebrospinal fluid (CSF) [13, 14]. Many studies have reported differences in the CSF α-synuclein levels between PD and control [15–17] as well as the diagnostic differentiation of different neurodegenerative diseases [18, 19]. However, the potential role of CSF α-synuclein as a biomarker for the presymptomatic phase of AD remains unclear.

In this study, we explored the associations between CSF α-synuclein and other AD biomarkers in the non-demented Chinese elderly. We also tested whether CSF α-synuclein was altered in patients with AD and with different pathophysiological profiles of AD based on the “ATN” classifications, and its associations with other AD biomarkers, cognitive decline and imaging evidence of neurodegeneration in the Alzheimer’s Disease Neuroimaging Initiative (ADNI) database. The value of CSF α-synuclein as a predictor of disease progression and neurodegeneration at the presymptomatic stage of AD was also investigated.

## Methods

### Study participants

Six hundred and fifty-one non-demented participants were from the Chinese Alzheimer’s Biomarker and Lifestyle (CABLE) study. The CABLE is a large-cohort study mainly focusing on Alzheimer’s risk factors and biomarkers in Chinese elderly adults. The participants in the CABLE study were recruited at Qingdao Municipal Hospital, consisting of cognitively normal (CN) and mild cognitive impairment (MCI) individuals. All participants were Han Chinese in origin and aged 50–90 years. The controls had Mini-Mental State Examination (MMSE) scores of 24 or higher, with lower scores indicating more impairment and higher scores less impairment (range, 0–30), and a Clinical Dementia Rating (CDR) score of 0, where lower scores indicate less impairment and higher scores more impairment (range, 0–3). The patients with MCI had MMSE scores of 24 or higher, an objective memory loss tested by delayed recall of the Wechsler Memory Scale (WMS) logical memory II (> 1 SD below the normal mean), a CDR score of 0.5, preserved activities of daily living, and absence of dementia. The exclusion criteria were: (1) central nervous system infection, head trauma, epilepsy, multiple sclerosis or other major neurological disorders; (2) major psychological disorders (e.g., depression); (3) severe systemic diseases (e.g., malignant tumors) that may affect CSF or blood levels of AD biomarkers including Aβ and tau; and (4) family history of genetic disease. All participants underwent clinical and neuropsychological assessments, biochemical testing, as well as blood and CSF sample collection. Demographic information, AD risk factor profile and medical history were also collected by a comprehensive questionnaire and an electronic medical record system.

Data were obtained from the ADNI database (adni.loni.usc.edu), an independent replication cohort. The ADNI was launched in 2003 as a public-private partnership under the leadership by Michael W. Weiner, MD, with a primary goal to test whether magnetic resonance imaging (MRI), positron emission tomography (PET), biological markers, as well as clinical and neuropsychological assessment can be combined to measure the progression of MCI and early AD. For up-to-date information, see www.adni-info.org.

Our ADNI cohort included all the CN controls, MCI patients and AD patients with available baseline samples for CSF α-synuclein. The inclusion/exclusion criteria are described at http://www.adni-info.org. In our study, we stratified the MCI group into stable MCI (sMCI) with no progression to AD dementia during at least 2-year follow-up, and progressive MCI (pMCI) with progression to AD dementia during at least 2-year follow-up. As a result, totally 4 groups were included: CN control, sMCI group, pMCI group and AD group. As to the “ATN” binary (i.e., positive or negative) categories, amyloid positive (A+) and negative (A-) were separated by a cutoff value of 192 pg/ml for CSF Aβ level; Tau pathology positive (T+) and negative (T-) were separated by a cutoff value of 23 pg/ml for CSF phosphorylated tau (p-tau) level.

The CABLE study was approved by the Institutional Ethics Committee of Qingdao Municipal Hospital. Written informed consent was obtained from all study participants directly or from their guardians. The ADNI study was approved by the Institutional Review Board at each of the participating centers, and all participants provided written informed consent.

### CSF/plasma biomarker measurements

CSF was collected by lumbar puncture through the L3/L4 interspace and gently mixed to avoid gradient effects. The samples were then centrifuged at 2000 g for 10 min to remove cells and other insoluble materials, stored in 1-ml aliquots at − 80°C until use for Aβ and tau analysis. CSF was sampled between 08:00 and 09:00 in the morning taking into account the possible circadian rhythm effect.

In the CABLE study, the concentrations of CSF Aβ42, total tau (t-tau), p-tau and CSF total α-synuclein were measured separately using an enzyme-linked immunosorbent assay (ELISA) kit (LEGEND MAX™ Human α-Synuclein ELISA Kit with pre-coated plate, Catalog No:844101), according to the manufacturer’s instructions. The samples and standards were measured in duplicate to generate an average value for the statistical analyses.

In the ADNI database, CSF Aβ_42_, t-tau and p-tau were measured at the ADNI biomarker core (University of Pennsylvania) using the multiplex xMAP Luminex platform (Luminex Corp, Austin, TX, USA) with the INNOBIA AlzBio3 kit (Fujirebio, Ghent, Belgium). The CSF neurofilament light chain (NFL) concentrations were measured using a commercial ELISA kit (Uman Diagnostics). The plasma NFL concentrations were measured using an NFL kit (NF-light; Uman Diagnostics), transferred onto the ultrasensitive single-molecule array platform using a home brew kit (Simoa Homebrew Assay Development Kit; Quanterix Corporation). The levels of CSF total α-synuclein concentrations in the ADNI cohort were measured by the Luminex MicroPlex Microspheres (Luminex Corp, Austin, TX), using the biotinylated goat anti-human α-syn antibody (R&D systems, Minneapolis, MN) as the detection antibody. The α-synuclein Luminex assay demonstrated a low day-to-day and plate-to-plate signal variety. The accuracy of the assay was further determined by the recovery of spiked α-synuclein protein, which was close to 93%.

### Neuroimaging

Structural MRI was performed only in the ADNI participants using a Siemens Trio 3.0 T scanner or Vision 1.5 T scanner (GE, Siemens and Philips). The regional volume estimates for the 1.5 and 3.0 T MRI images were processed with the Free-surfer software package version 4.3 and 5.1 image processing frameworks, respectively. The hippocampus and ventricles were selected as the regions of interest.

### Statistical analyses

The associations between CSF α-synuclein and demographic factors were analyzed with the Mann-Whitney test and the Spearman rank correlation test. The associations of CSF α-synuclein with CSF Aβ42, t-tau, and p-tau levels were analyzed with the linear regression after adjustment for age, gender, educational level, diagnosis and *APOE* ε4 genotype (with CSF α-synuclein as a predictor). In the ADNI database, associations between CSF α-synuclein concentrations and the diagnostic groups were tested in an analysis of covariance model adjusted for age, gender, educational level and *APOE* ε4 genotype. The effect of different CSF analytes on the risk of conversion to AD was assessed with the logistic regression analysis. The receiver-operator curves and the area under the curves were derived from the predictive probabilities of the logistic regression models. We tested the associations of CSF α-synuclein concentrations with longitudinal cognition and brain structure using the linear mixed-effects models. These models had random intercepts and slopes for time and an unstructured covariance matrix for the random effects and included the interaction between (continuous) time and CSF α-synuclein as predictor with adjustment for confounders. All tests were 2-sided. Statistical significance was set at *P* < 0.05. All regression analyses were corrected for age, gender, educational level, diagnosis, and *APOE* ε4 genotype. The following variables were natural log-transformed to ensure normality: CSF α-synuclein, p-tau, t-tau, and Aβ levels, and hippocampus volume. All statistical analyses were performed using R version 3.4.0 (R Foundation).

## Results

### Characteristics of participants in the CABLE study

We included 651 non-demented elders from the CABLE study, consisting of 457 CN controls (238 females, 60.54 ± 10.46 years) and 194 MCI patients (109 females, 63.6 ± 9.72 years) (Table 1). The CN individuals were significantly younger and more educated, and had significantly lower levels of CSF p-tau and t-tau, compared to the MCI participants.

**Table 1:** Demographics of the study population in CABLE ^a^

	CN (*n* = 457)	MCI (*n* = 194)	*P* value
AGE, mean (SD), years	60.93 (10.55)	65.44 (10.01)	< 0.001
Female, *n* (%)	269 (58.9)	109 (56.2)	0.59
APOE ε4 genotype carriers, *n* (%)	69 (15.1)	35 (18.0)	0.41
Education, mean (SD), years	10.38 (6.12)	8.56 (4.23)	< 0.001
CSF α-synuclein, mean (SD), ng/l	1466.73 (813.99)	1501.19 (914.13)	0.61
CSF p-tau, mean (SD), ng/l	38.11 (9.69)	40.04 (12.42)	0.03
CSF t-tau, mean (SD), ng/l	173.3 (77.96)	191.02 (122.57)	0.03
CSF Aβ_42_, mean (SD), ng/l	160.01 (91.51)	162.10 (105.53)	0.81

*Aβ* β-amyloid, *CN* Cognitively normal, *CSF* Cerebrospinal fluid, *MCI* Mild cognitive impairment, *MMSE* Mini-Mental State Examination, *p-tau* Phosphorylated tau, *t-tau* Total tau.

^a^*P* values from the Kruskal-Wallis test or Fisher exact test

### CSF α-synuclein and established AD biomarkers in the CABLE study

In the CABLE study, we examined the concentrations of CSF α-synuclein and other established AD biomarkers (CSF Aβ, p-tau and t-tau) and tested their relationships (Table 2). We found that the level of CSF α-synuclein was positively associated with the CSF t-tau (β = 0.56, *P* < 0.001) and p-tau (β = 0.35, *P* < 0.001) among the non-demented participants. However, there was no association between CSF α-synuclein and CSF Aβ level at baseline. In addition, the same associations were found in the CN group and the MCI group (Table 2).

**Table 2:** Correlations between CSF α-synuclein and other biochemical markers in CABLE ^a^

	All participants		CN		MCI	
	β coefficient	*P* value	β coefficient	*P* value	β coefficient	*P* value
CSF t-tau	0.56	< 0.001	0.38	< 0.001	0.67	< 0.001
CSF p-tau	0.35	< 0.001	0.27	< 0.001	0.40	< 0.001
CSF Aβ42	−0.02	0.97	−0.01	0.82	−0.07	0.69

*Aβ* β-amyloid, *CABLE* Chinese Alzheimer’s Biomarker and Lifestyle, *CN* Cognitively normal, *CSF* Cerebrospinal fluid, *MCI* Mild cognitive impairment, *p-tau* Phosphorylated tau, *t-tau* Total tau.

^a^Data are β coefficients (with *P* values) from linear regression models for correlations between CSF α-synuclein and other biomarkers, adjusted for age, gender, educational level and *APOE* ε4 genotype. Models were tested in the whole cohort and in individual diagnostic groups

### Characteristics of participants in ADNI

Three hundred and eighty-two subjects from the ADNI database were included (Table 3). This cohort consisted of 109 CN controls (54 females, 75.63 ± 5.22 years), 117 sMCI patients (37 females, 74.34 ± 7.60 years), 66 pMCI patients (25 females, 74.21 ± 7.58 years) and 90 AD patients (39 females, 74.89 ± 7.72 years). According to the new “ATN” scheme, 258 A+ (220 A + T+) patients and 124 A- (96 A-T-) controls were included. As expected, the AD group had the highest frequency of the *APOE* ε4 allele (69.23%) and the CN group had the lowest frequency (23.85%). There was no significant difference in the educational level (*P* = 0.16) or age (*P* = 0.53) among these four groups. Furthermore, AD patients had lower MMSE scores compared with the MCI patients and CN controls (*P* < 0.01).

**Table 3:** Demographics of the study population in ADNI

	CN (*n* = 109)	sMCI (*n* = 117)	pMCI (*n* = 66)	AD (*n* = 90)
Age, mean (SD), years	75.63 (5.22)	74.34 (7.60)	74.21 (7.58)	74.89 (7.72)
Female, *n* (%)	54 (49.54)	37 (31.62)	25 (36.76)	39 (44.32)
*APOE* ε4 genotype carriers, *n* (%)	26 (23.85)	55 (47.00)	42 (61.76)	63 (69.23)
CSF α-synuclein, mean (SD), ng/L	0.46 (0.17)	0.54 (0.22)	0.56 (0.20)	0.61 (0.24)
MMSE score, mean (SD)	29.07 (1.05)	27.15 (1.64)	26.58 (1.77)	23.39 (1.80)
CSF Aβ42, mean (SD), ng/L	208.70 (52.36)	174.69 (55.28)	148.75 (41.52)	143.99 (38.31)
CSF t-tau, mean (SD), ng/L	69.08 (29.85)	97.31 (64.77)	112.00 (41.52)	122.83 (57.09)
CSF p-tau, mean (SD), ng/L	25.04 (13.93)	32.76 (18.31)	39.50 (17.18)	41.48 (19.73)
Hippocampus volume, mm^3^	6648.16 (766.59)	5964.07 (986.76)	5522.46 (1044.15)	5217.39 (1043.40)

Abbreviations: *Aβ* β-amyloid, *AD* Alzheimer disease dementia, *CN* Cognitively normal, *CSF* Cerebrospinal fluid, *sMCI* Stable mild cognitive impairment, *pMCI* Progressive mild cognitive impairment, *MMSE* Mini-Mental State Examination, *p-tau* Phosphorylated tau, *t-tau* Total tau

### CSF α-synuclein and established AD biomarkers in ADNI

In the ADNI database, we found that the high CSF α-synuclein levels were associated with the high CSF t-tau (β = 0.27, *P* < 0.001) and p-tau (β = 0.36, *P* < 0.001) in the whole cohort. However, there was no association between CSF α-synuclein and CSF Aβ level at baseline. The same results were obtained in the MCI group (CSF t-tau: β = 0.29, *P* < 0.001, CSF p-tau: β = 0.33, *P* < 0.001) and CN controls (CSF t-tau: β = 0.2, *P* < 0.001, CSF p-tau: β = 0.32, *P* < 0.001). In addition, the CSF α-synuclein concentration was associated with CSF NFL concentration in non-demented elders (β = 0.12, *P* < 0.001). However, there was no association between CSF α-synuclein and plasma NFL (Table 4, Fig. S1).

**Table 4:** Modelling the association of CSF biomarkers on AD biomarkers and clinical outcomes in ADNI^a^

	All Participants		MCI		CN	
Cross-sectional (MR)	β coefficient	*P* value	β coefficient	*P* value	β coefficient	*P* value
CSF t-tau	0.27	< 0.001	0.29	< 0.001	0.20	< 0.001
CSF p-tau	0.36	< 0.001	0.33	< 0.001	0.32	< 0.001
CSF Aβ42	−0.03	0.33	−0.04	0.32	0.006	0.86
CSF NFL	0.12	< 0.001	0.11	0.04	0.03	0.45
Plasma NFL	0.04	0.27	0.02	0.73	−0.04	0.53
Longitudinal (MELM)						
Hippocampus	−0.008	0.001	−0.007	0.04	−0.003	0.17
Ventricles	0.006	0.13	0.005	0.36	0.003	0.43
Cox (Hazard ratio)	**Statistic**	*P* value				
MCI-to-AD dementia conversion	1.53 (1.15–2.0)	0.004				

Abbreviations: *CN* Cognitively normal, *CSF* Cerebrospinal fluid, *MCI* Mild cognitive impairment, *p-tau* Phosphorylated tau, *t-tau* Total tau, *Cox* Cox proportional hazards model, *MELM* Mixed effects linear model, *MR* Multiple regression

^a^All models were adjusted for age, gender, educational level, *APOE* ε4 genotype and intracranial volume (for MRI only). Models were tested in the whole cohort and in individual diagnostic groups

### CSF α-synuclein in different diagnostic groups in ADNI

**Fig. 1:**
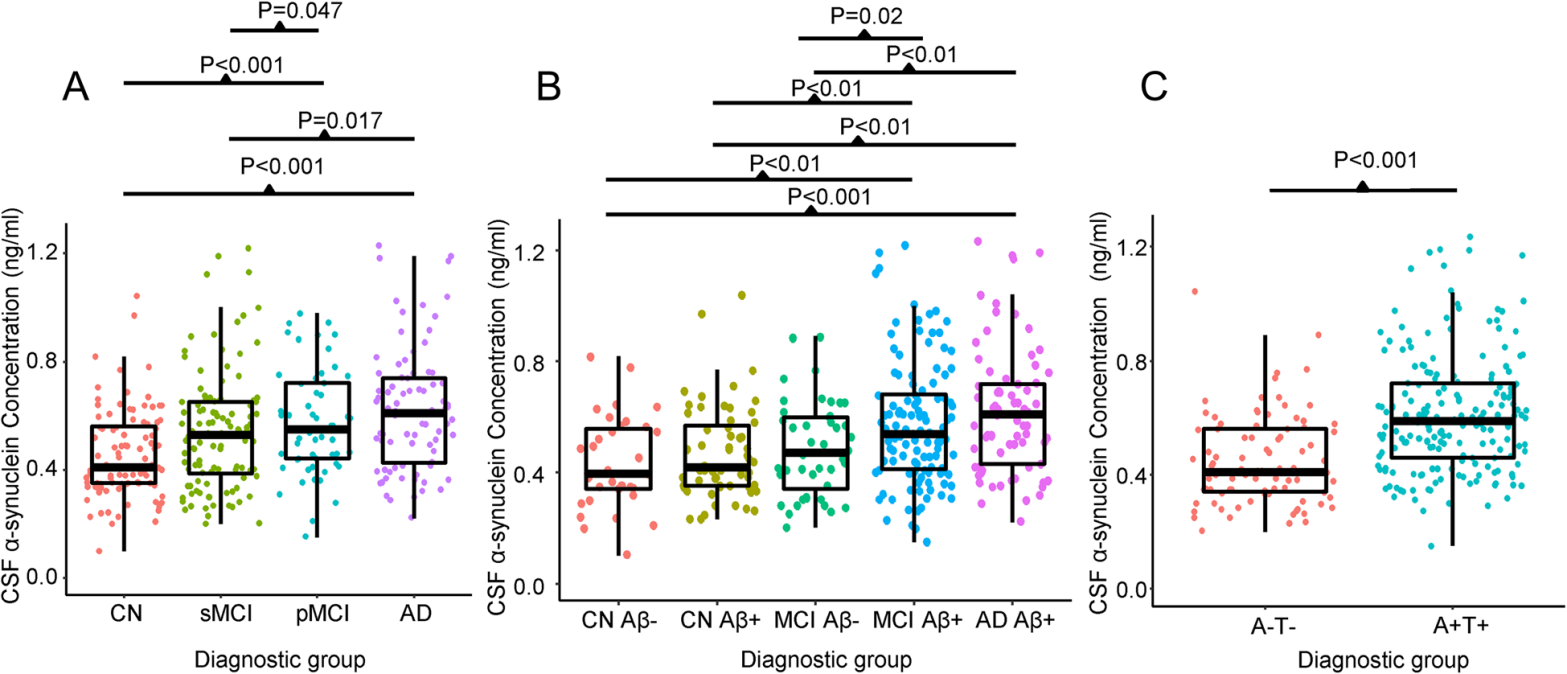
Scatter plots of cerebrospinal fluid α-synuclein concentrations in the diagnostic groups. The colors of the scatterplots are grouped by different diagnostic groups. The three horizontal black lines in each boxplot indicate the median and interquartile range. The whiskers extend to the minimum and maximum CSF α-synuclein data points. **a** CSF α-synuclein concentration in the diagnostic groups. **b** CSF α-synuclein concentration in the diagnostic groups stratified by Aβ pathology. **c** CSF α-synuclein concentration in the AD pathophysiology (tau and amyloid-β) positive and negative subgroups. A-, Aβ negative; A+, Aβ positive; T-, tau negative; T+, tau positive

The level of CSF α-synuclein showed a trend of increase with the progression of disease stage. The CSF α-synuclein concentration was significantly higher in the AD and pMCI groups than in the CN controls (*P* < 0.0001 and *P* < 0.001, respectively) and the sMCI group (*P* = 0.02 and *P* = 0.04, respectively) (Fig. 1a). In addition, the A+ AD group had higher CSF α-synuclein levels than the A- controls (*P* < 0.001), A+ controls (*P* < 0.001), and A- MCI group (*P* < 0.001) (Fig. 1b). The A+ MCI had higher CSF α-synuclein levels than the A- controls (*P* < 0.01), A+ controls (*P* < 0.01), and the A- MCI group (*P* = 0.02). The CSF α-synuclein level was also significantly different between the A + T+ group and the A-T- group (*P* < 0.0001) (Fig. 1c).

We generated receiver-operating curves based on the logistic regression models adjusted for age at baseline, gender, educational level and *APOE* ε4 genotype to assess the predictive value of CSF α-synuclein alone and in combination with other established AD biomarkers for the risk of conversion to AD. The area under the curve (AUC) of the baseline model containing CSF α-synuclein, age at baseline, gender, educational level and *APOE ε4* genotype was 0.76 in predicting the onset of AD among the CN controls, and the AUC was further increased by the inclusion of CSF tau/Aβ ratio (AUC = 0.88) (Fig. S2). As expected, the baseline model showed a similar predicting value for the onset of pMCI among the CN controls (Fig. S3). In the A- group, this baseline model showed a good predictive value for the risk of conversion to A+ status (AUC = 0.77), and inclusion of CSF t-tau (AUC = 0.88) and p-tau (AUC = 0.92) further enhanced this predictive value (Fig. S4). Furthermore, the baseline model performed best when the participants were grouped by Aβ deposition and pathology (AUC = 0.84). We also detected that CSF α-synuclein added value for diagnosis prediction (Fig. S5).

### CSF α-synuclein, longitudinal neuroimaging change and progression in ADNI

**Fig. 2:**
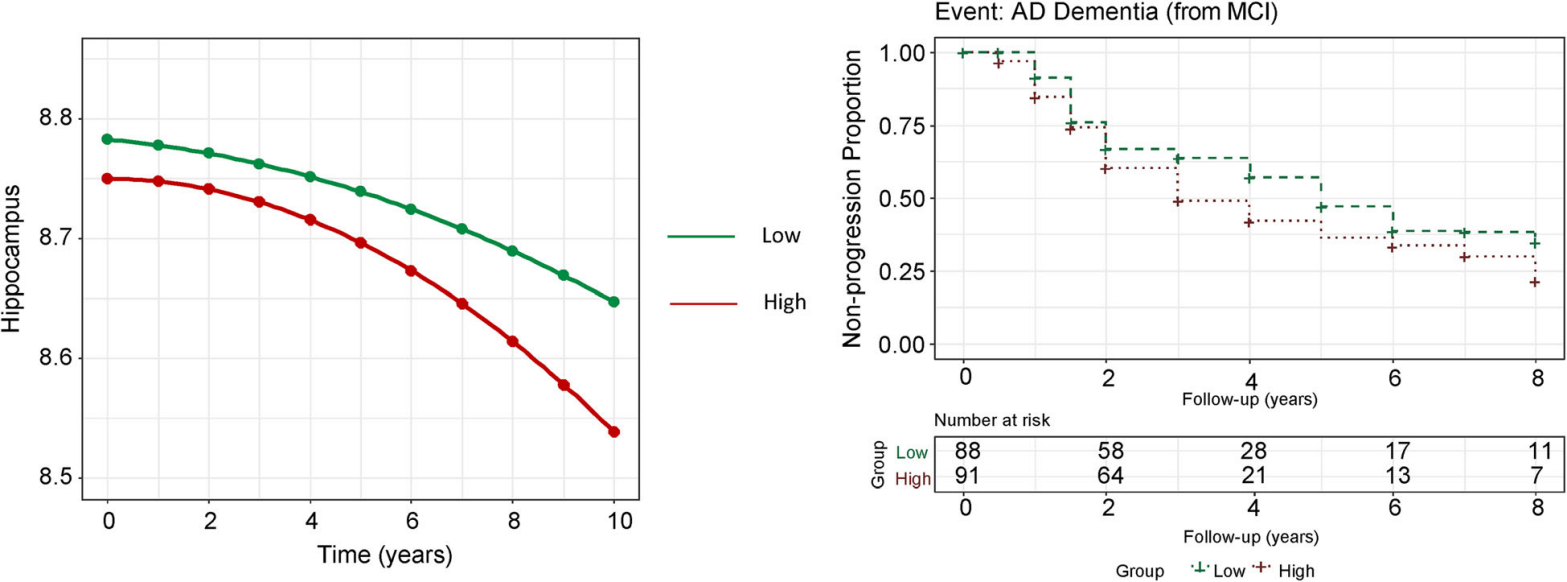
Associations between CSF α-synuclein and longitudinal neuroimaging change in ADNI. Data from linear mixed-effects models (left) and cox proportional hazards models (right) were adjusted for age, gender, educational level and *APOE* ε4 genotype. Hippocampal volume on the y axis was log-transformed to ensure normality. Time (years) on the x axis indicated follow-up years, in which “0” indicating the baseline. All participants were classified into High and Low groups according to their baseline CSF α-synuclein concentration (High: greater than median; Low: less than median)

Next, the linear mixed-effects models were utilized to test the associations between baseline CSF α-synuclein concentration and subsequent disease progression, after adjustment for age, gender, educational level, diagnosis, and *APOE* ε4 genotype. The baseline CSF α-synuclein concentration was found to be significantly associated with the hippocampal volume (β = − 0.008, *P* = 0.001 longitudinally) (Table 4, Fig. 2 (left)).

Figure 2 (right) presents the results of Kaplan-Meier analysis. The cox proportional hazards model was developed to estimate the predictive value of CSF α-synuclein for the conversion risk from MCI to incidence of AD dementia, after controlling for baseline age, gender and years of education. MCI individuals with high CSF α-synuclein levels would satisfy the diagnostic criteria for AD at a comparatively earlier interval (hazard ratio 2.79, 95% CI 1.14–6.9, *P* = 0.03) (Table 4).

## Discussion

In this study, we found that the CSF α-synuclein concentration (1) was associated with CSF t-tau and p-tau levels among the non-demented elderly adults, (2) was elevated in the AD dementia group and the Aβ/tau-positive group compared with the control group, and (3) could predict hippocampal atrophy and the conversion from MCI to AD dementia. Taken together, these findings suggest that CSF α-synuclein is a very early and potentially presymptomatic biomarker for AD. This biomarker may be helpful for AD diagnosis and prediction of disease progression and staging of AD even in the preclinical stage.

“Pure” AD is characterized by the presence of both diffused neuritic plaques and intracellular neurofibrillary tangles, with a lack of abnormal α-synuclein inclusions or neuritis. However, more than 50% of AD patients exhibit excessive brain accumulation of α-synuclein-positive Lewy bodies, particularly in the amygdala [10, 20]. The presence of α-synuclein seems not to be innocuous, as these patients demonstrate an accelerated cognitive decline than subjects with AD alone [12, 21]. Previous studies have indicated that α-synuclein can be secreted into the surrounding media in the brain and then to the CSF [22, 23]. Therefore, the CSF could be used to investigate the mechanisms of α-synuclein metabolism in the brain.

Consistent with most studies, our study showed that CSF α-synuclein was higher in the AD group compared with the CN controls and MCI group. A possible hypothesis is that the higher level of α-synuclein could induce a decrease in some proteins in synaptic vesicle and alterations of the protein composition of synaptic vesicles, thus causing neuronal damage in AD, which in turn increases the release of α-synuclein from damaged cells into the CSF [24, 25]. As the CSF α-synuclein levels are lower in synucleinopathies compared to control, but appear higher in AD/MCI than control, the α-synuclein may serve as a biomarker for differential dementia diagnosis. In this study, logistic regression analysis was used to assess the effect of CSF analytes on the risk of progression to AD. The AUC (which reflects the predictive probabilities of the logistic regression models) of the model including CSF α-synuclein, age at baseline, gender, educational level and *APOE* ε4 genotype had good performance in predicting progression from CN to pMCI or AD. Recently, the NIA-AA committee has recommended a different definition of AD by pathophysiology, independent of the clinical symptoms. They proposed that as long as biomarker evidence of Aβ and tau pathology was present simultaneously, the term “Alzheimer’s disease” would be applied. Here the CSF α-synuclein model had high diagnostic accuracy for patients with the diagnosis of AD based on the “ATN” system (A + T+) vs controls (A-T-) (AUC = 0.84, which was comparable to other established CSF biomarkers).

Many lines of evidence have suggested that the pathological α-synuclein, Aβ and tau have synergistic adverse effects to promote the aggregation of each other, thereby amplifying the neuronal damage [24, 26–31]. Notably, α-synuclein inclusions are commonly observed in patients with familial Down’s syndrome, where Aβ peptides are highly expressed. In both diseases, α-synuclein affects the biological pathways and promotes the formation of Aβ aggregates. α-Synuclein has also been proposed to be implicated in synaptic vesicle formation, axonal transport as well as dopamine synthesis and metabolism [32]. In normal conditions, the synaptic membrane is integrated and the α-synuclein is completely released into the cytosol. However, in the event of neuronal damage and synaptic membrane defect, both aggregated Aβ and α-synuclein might attach to synaptic membrane and accumulate in lipid rafts. The synaptic membrane-bound α-synuclein could not only induce cytosolic α-synuclein to aggregate as intracellular Lewy bodies but also interact with the membrane-associated Aβ_40_ and Aβ_42_ peptides [33]. This may explain the low level of CSF α-synuclein in individuals with normal cognitive function to a certain extent. Moreover, an in vitro experiment has demonstrated that the interaction with Aβ_1–42_ is sufficient to induce the intracellular accumulation of α-synuclein, whereas interaction with Aβ_1–40_ is not [34]. In our study, however, we did not find any association between CSF α-synuclein and CSF Aβ levels at baseline. The reason may be that this mutual effect occurs in the initial stages of the mixed pathology, preceding the presence of intracellular α-synuclein in surrounding media and eventually in the CSF by years or decades. We only studied the CSF total α-synuclein level rather than the oligomeric or phosphorylated forms. Future studies focusing on the oligomeric or phosphorylated forms of α-synuclein may provide additional information.

Moreover, α-synuclein has also being observed in progressive supranuclear palsy [35] and frontotemporal dementia [36]. Many studies have proposed that α-synuclein and tau interact to promote the fibrillation and toxicity of each other [26]. However, unlike α-synuclein that could spontaneously polymerize into amyloidogenic fibrils, tau requires cofactors such as glycosaminoglycans or nucleic acids to polymerize [37]. The α-synuclein polymers act as amyloidogenic “seeds” or as amyloidogenic chaperones that induce the formation of tau fibrillary inclusions even in the absence of α-synuclein coexpression [26, 27, 38]. Besides, Tau promotes α-synuclein to polymerize into fibrils. Low concentrations of α-synuclein do not fibrillize without tau, however, in the presence of tau, most α-synuclein assembles into fibrils. Much attention has been paid to the relationship between CSF α-synuclein and tau. Consistent with most studies [24, 29], our study found positive associations of CSF α-synuclein with CSF t-tau and p-tau levels in the CABLE study. We noted that the mean values for CSF α-synuclein and CSF Aβ levels between controls in the 2 Chinese cohorts using similar assays were different. This could partly be explained by the differences in pre-analytical protocols, analytical procedures, assay quality and the absolute levels between assay formats [39]. In addition, the CSF α-synuclein also correlated with the CSF NFL in the whole cohort, but not in the CN group, suggesting that they were confounded by diagnosis. This finding probably reflects that several different pathological conditions (e.g., degeneration of different types of axons) may drive the different biomarker responses. We also tested the association between CSF α-synuclein and plasma NFL concentration, but did not find any significant association. More studies with larger sample sizes are needed to clarify whether α-synuclein and NFL reflect the same neurodegeneration pattern.

Importantly, we found that the CSF α-synuclein levels might correlate with AD severity and progression, which was consistent with a recent study indicating that increased α-synuclein displayed a stronger association with cognitive impairment than soluble Aβ and tau levels [40]. It has been widely recognized that α-synuclein is a synaptic marker. α-Synuclein is highly expressed in the pre-synaptic terminals [41, 42] and plays a role in the regulation of neurotransmitter release, synaptic function and plasticity. It could trigger synaptoxicity not only by directly damaging the synaptic membrane, but also by damaging the mitochondria, lysosomes, or microtubules, leading to dendritic and spine alterations, axonal dystrophy, and eventually neuronal loss [43]. Along with the synaptic damage, α-synuclein is released into the CSF. Therefore, it is reasonable to assume that the CSF α-synuclein level correlates with cognitive decline in AD, since synaptic damage is supposed to be a strong predictor of cognitive decline [44].

## Conclusions

CSF α-synuclein was associated with CSF t-tau and p-tau levels among the non-demented elderly adults. In the ADNI database, CSF α-synuclein concentrations were increased with the severity of the disease. CSF α-synuclein predicted longitudinal hippocampus atrophy and conversion from MCI to AD dementia. The current findings suggest CSF α-synuclein as a very early and potentially presymptomatic biomarker for AD, a prognostic marker in the clinic, and an outcome measure in clinical trials.

## Supplementary Information

**Additional file 1: Supplementary Figure S1.** Association between CSF α-synuclein and CSF/plasma NFL concentration. Linear regression trend lines are shown in blue. These regression lines were unadjusted, while the corresponding analysis in the text was adjusted for age, sex, educational level, and *APOE* ε4 genotype. CSF/plasma NFL and CSF a-synuclein concentrations on the y- and x-axes were logarithmic.

**Additional file 2: Supplementary Figure S2.** Receiver operating curves to assess the diagnostic accuracy of CSF α-synuclein and other biomarkers for AD dementia. Receiver operating curves of logistic regression model were controlled for age at baseline, gender, educational level and *APOE* ε4 genotype.

**Additional file 3: Supplementary Figure S3.** Conversion from CN to pMCI as predicted by baseline CSF biomarkers. Receiver operating curves of the logistic regression model controlling for age at baseline, gender, educational level and *APOE* ε4 genotype for predicting conversion to pMCI among people with normal cognitive function.

**Additional file 4: Supplementary Figure S4.** Conversion from Aβ-negative status to Aβ-positive group as predicted by the baseline CSF biomarkers. Receiver operating curves of the logistic regression model were controlled for age at baseline, gender, educational level and *APOE* ε4 genotype for predicting conversion to Aβ-positive status among the Aβ-negative group.

**Additional file 5: Supplementary Figure S5.** Receiver operating curves for predicting conversion from AD pathophysiology (tau and amyloid-β) negative to positive. The baseline model included age at baseline, gender, educational level and *APOE* ε4 genotype.

## Data Availability

Not applicable.

## Abbreviations

ADNI: Alzheimer’s Disease Neuroimaging Initiative
Aβ: β-amyloid
AD: Alzheimer’s disease
CSF: Cerebrospinal fluid
CABLE: Chinese Alzheimer’s Biomarker and Life style
CN: Cognitively normal
CDR: Clinical Dementia Rating
DLB: Dementia with Lewy bodies
MMSE: Mini-Mental State Examination
MRI: Magnetic resonance imaging
MCI: Mild cognitive impairment
PET: Positron emission tomography
PD: Parkinson’s disease
