# Supplementary figures and images for: Cerebrospinal fluid α-synuclein predicts neurodegeneration and clinical progression in non-demented elders
Source: Transl Neurodegener. 2020 Nov 23;9:41. doi: 10.1186/s40035-020-00222-1 (PMC7685645; doi:10.1186/s40035-020-00222-1)

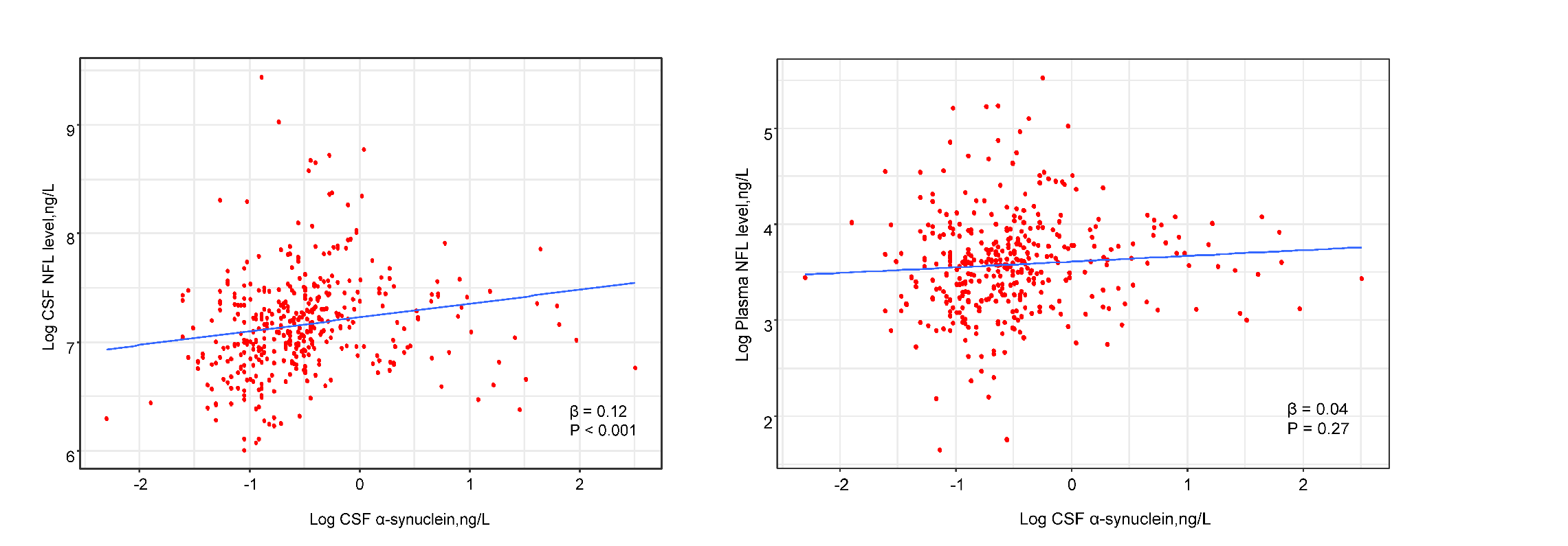

Supplement: Supplementary file 1 — Additional file 1: Supplementary Figure S1. Association between CSF α-synuclein and CSF/plasma NFL concentration. Linear regression trend lines are shown in blue. These regression lines were unadjusted, while the corresponding analysis in the text was adjusted for age, sex, educational level, and APOE ε4 genotype. CSF/plasma NFL and CSF a-synuclein concentrations on the y- and x-axes were logarithmic. [file 40035_2020_222_MOESM1_ESM.tif]

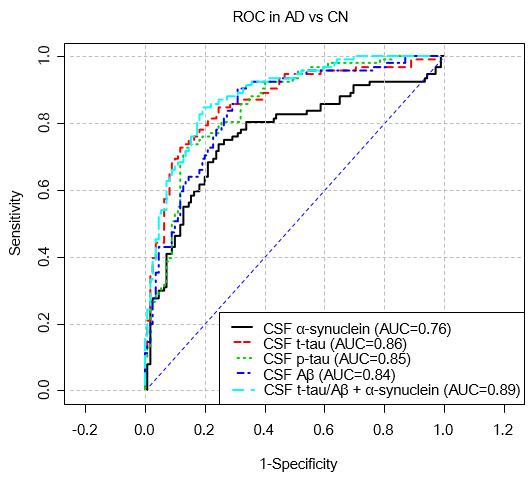

Supplement: Supplementary file 2 — Additional file 2: Supplementary Figure S2. Receiver operating curves to assess the diagnostic accuracy of CSF α-synuclein and other biomarkers for AD dementia. Receiver operating curves of logistic regression model were controlled for age at baseline, gender, educational level and APOE ε4 genotype. [file 40035_2020_222_MOESM2_ESM.tif]

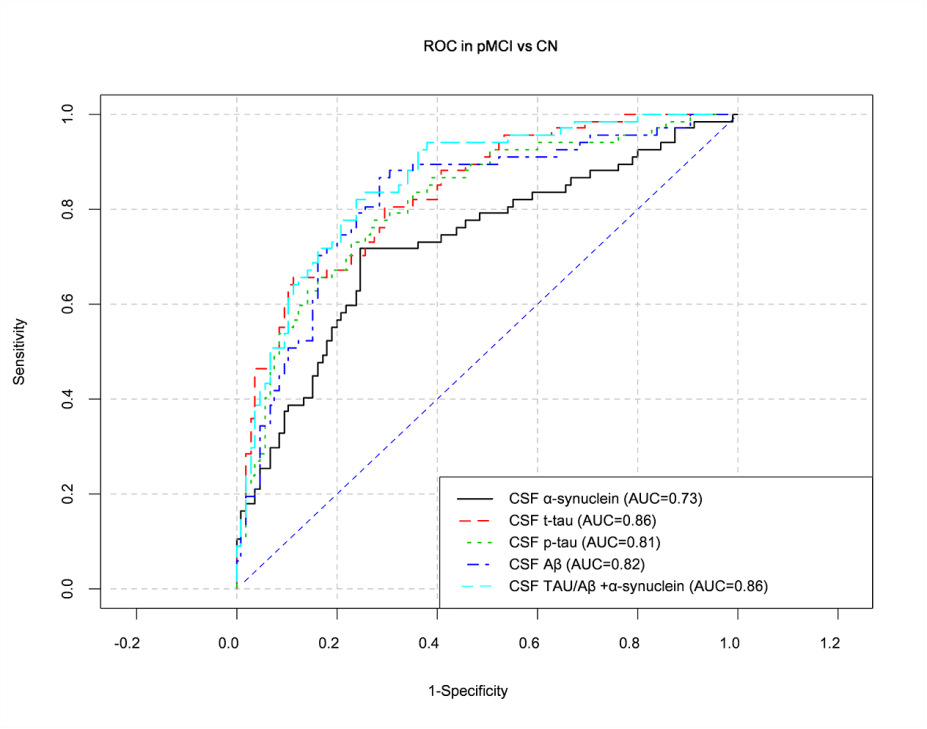

Supplement: Supplementary file 3 — Additional file 3: Supplementary Figure S3. Conversion from CN to pMCI as predicted by baseline CSF biomarkers. Receiver operating curves of the logistic regression model controlling for age at baseline, gender, educational level and APOE ε4 genotype for predicting conversion to pMCI among people with normal cognitive function. [file 40035_2020_222_MOESM3_ESM.tif]

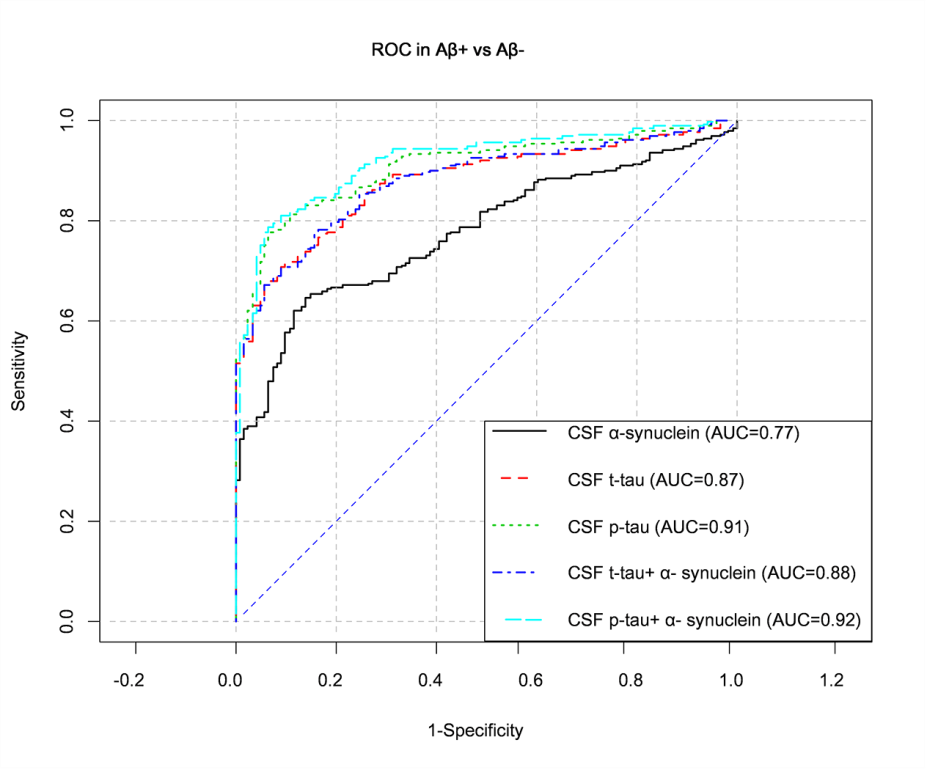

Supplement: Supplementary file 4 — Additional file 4: Supplementary Figure S4. Conversion from Aβ-negative status to Aβ-positive group as predicted by the baseline CSF biomarkers. Receiver operating curves of the logistic regression model were controlled for age at baseline, gender, educational level and APOE ε4 genotype for predicting conversion to Aβ-positive status among the Aβ-negative group. [file 40035_2020_222_MOESM4_ESM.tif]

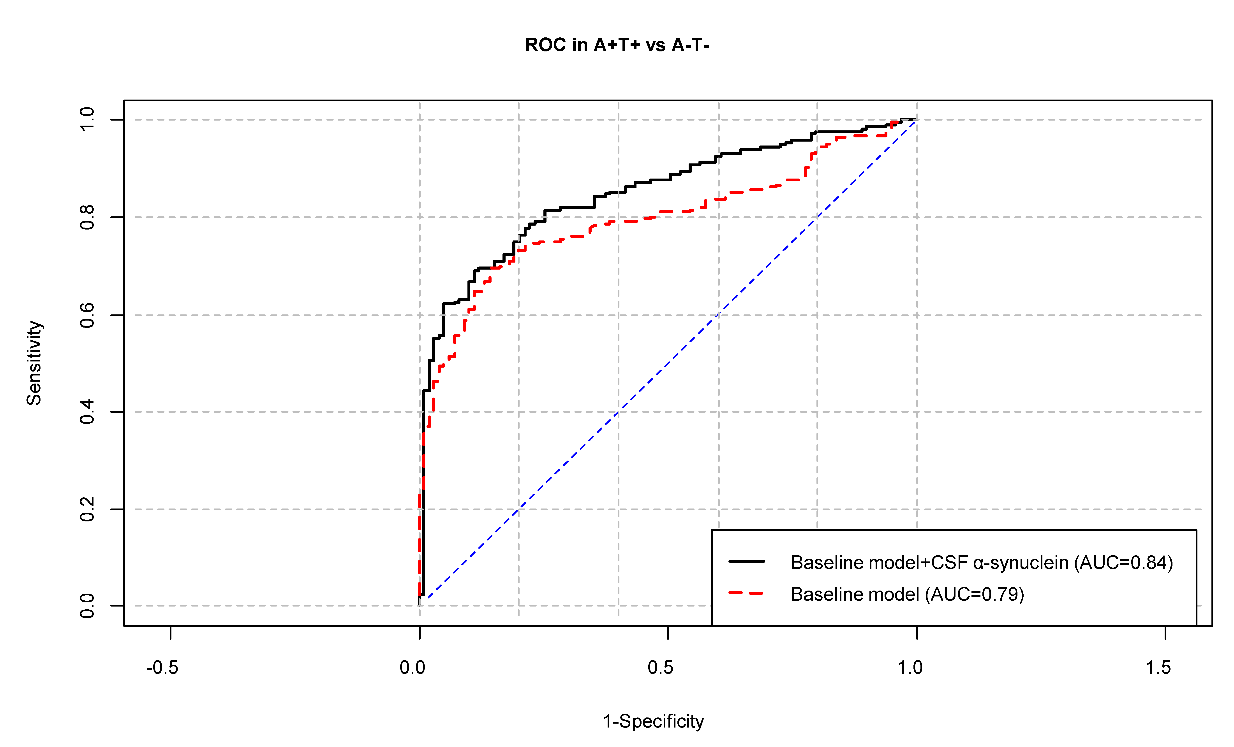

Supplement: Supplementary file 5 — Additional file 5: Supplementary Figure S5. Receiver operating curves for predicting conversion from AD pathophysiology (tau and amyloid-β) negative to positive. The baseline model included age at baseline, gender, educational level and APOE ε4 genotype. [file 40035_2020_222_MOESM5_ESM.tif]
